# Physico-Chemical Properties of NaV_3_O_8_ Prepared by Solid-State Reaction

**DOI:** 10.3390/ma14226976

**Published:** 2021-11-18

**Authors:** Mariya Shchelkanova, Georgiy Shekhtman, Svetlana Pershina, Emma Vovkotrub

**Affiliations:** Institute of High Temperature Electrochemistry, Ural Branch, Russian Academy of Sciences, 20 Akademicheskaya St., 620990 Ekatherinburg, Russia; shekhtman@ihte.uran.ru (G.S.); SVPershina_86@mail.ru (S.P.); E.Vovkotrub@ihte.uran.ru (E.V.)

**Keywords:** sodium power sources, cathode materials, sodium–vanadium oxides, solid state synthesis

## Abstract

Sodium–vanadium oxide NaV_3_O_8_ is synthesized via solid-state method and optimum synthesis conditions are chosen based on the data of DSC and TG analysis. The material synthesized is characterized by X-ray phase analysis, Raman spectroscopy and scanning electron microscopy. The ratio V^4+^/V^5+^ in the sample obtained is determined by X-ray photoelectron spectroscopy. Conductivity of the material synthesized was measured by impedance spectroscopy, pulse potentiometry and DC method over the range RT–570 °C. It is shown that NaV_3_O_8_ has rather high conductivity essentially electron in type (6.3 × 10^−2^ at room temperature). AC and DC conductivity measurements are performed and cycling of symmetricNaV_3_O_8_|Na_3.85_Zr_1.85_Nb_0.15_Si_3_O_12_|NaV_3_O_8_ cell in galvanostatic conditions. Thermal stability is studied across 25–570 °C temperature range. The results obtained are compared with the properties of NaV_3_O_8_ produced via aqueous solution.

## 1. Introduction

Lithium and lithium-ion batteries (LIBs) rank high among electrochemical power sources nowadays, because they deliver high specific energy and the highest working voltage. High-energy lithium-ion batteries show successful applications in low power equipment, such as consumer electronics. However, Li belongs to the rare metals, so the possibilities of extending the use of lithium-ion batteries to large equipment for power industry and electric vehicles will be restricted by the low availability of lithium raw materials. In this area, there is currently a lot of interest in developing power sources having characteristics close to those of LIBs but using easily available low-cost materials. Sodium and sodium-ion batteries (SIBs) are considered as materials that meet these requirements [1,2,3].

Sodium has a slightlysmaller negative potential than lithium (−2.71 V versus −3.03 V), but at the same time it has a number of substantial advantages, the chief one being its almost inexhaustible resources in nature, easy regeneration and, as consequence, a lower cost of sodium batteries compared to lithium ones. Additionally, the majority of power sources with alkali-metal anodes use solutions of some inorganic salts in organic liquids as electrolytes. Such liquids areusually highly flammable, which creates safety issues due to a potential electrolyte leakage followed by its explosion or ignition. In this connection, moving away from liquid electrolytes and transition to all-solid-state power sources seems to be a very attractive option [4,5]. Solid electrolytes used in all-solid-state power sources are generally characterized by low conductivity; therefore, such power sources will deliver the best performance in devices operating at elevated temperatures. In view of this, designing efficient cathode materials stable at elevated temperatures (~300 °C) isone of the basic areas in the development of all-solid-state power sources.

Application of vanadium oxides V_m_O_n_ (V_2_O_5_, V_6_O_13_, V_6_O_14_) [6] and their derivatives [7,8] as cathode materials of power sources has recently become the subject of intensive research. Both vanadium oxides and alkali metal vanadates are characterized by a layered structure formed by layers of vanadium–oxygen polyhedra [9]. There is enough space between the layers to allow the insertion of various cations, monovalent as well as polyvalent, which means that such compounds are capable of reversible intercalation of different ions. V–O bonds between the vanadium–oxygen layers are weak, which allows the layers to shift against each other and additionally increases the maximum possible amount of intercalated cations, i.e., the intercalation capacity of the material. The high theoretical capacity and energy density of vanadium oxides make them attractive cathode materials for power sources. Moreover, vanadium oxides and alkali metal vanadates often contain vanadium in lower oxidation states alongside V^5+^ ions, which generates quite considerable electronic conductivity and, consequently, enhances the characteristics of the cathode material. Finally, vanadium oxides and their derivatives have an advantage of a relatively low cost. Thus, owing to the above-mentioned characteristics, vanadium oxides and alkali metal vanadates are attractive as cathode materials for power sources and are currently a subject of intensive research.

Cathodes based on vanadium oxides initially showed good performance in lithium and lithium-ion power sources [10,11]. Subsequently, with the growing interest in sodium power sources, sodium–vanadium oxides were proposed as cathode materials to be used in such batteries [12]. Studies into Na batteries with cathodes based on NaV_6_O_15_ [13], Na_0.33_V_2_O_5_ [14] and Na_1.5+y_VO_3_ [15] confirmed the high potential of such materials and gave reason to continue investigating the compounds under discussion in terms of their possible application as active cathode materials of SIBs.

NaV_3_O_8_ is one of sodium–vanadium compounds currently attracting considerable scientific interest as a potential cathode material for sodium and sodium-ion power sources [16,17,18,19]. According to the literature data, Na-ion power sources with NaV_3_O_8_ cathode show a good performance in terms of specific energy. Thus, a cell with a sodium anode, an electrolyte of NaClO_4_ solution in PC and a cathode containing NaV_3_O_8_ nanorods as active material delivered the reversible capacity of 162.1 mA·h·g^−1^ and coulombic efficiency of 96% under the current density of 120 mA·g^−1^ during 500 charge/discharge cycles. The reversible capacity of 88 mA·h·g^−1^ is retained even at elevated current densities of about 2 A·g^−1^ [15].

The available literature data indicate that the electrical characteristics of power sources with NaV_3_O_8_-based cathodes depend to a great extent on the morphology, particle size, crystallinity degree of the active cathode material and other factors, which, in their turn, depend on the technique by which the vanadate was initially synthesized [12]. Various methods of producing NaV_3_O_8_ have been proposed in the literature, e.g., it can be synthesized in the form of nanobelts [17], nanorods [19], or core/shell type nanoparticles [20], but these methods are long and complicated. In view of this, research into the physico-chemical properties and electrochemical behavior of NaV_3_O_8_ obtained through simple, traditional techniques is, no doubt, of great interest. Previouslywehave performed such experiments for NaV_3_O_8_ obtained by the method of aqueous solution [21]. Here we report on the results for NaV_3_O_8_ produced via solid-state synthesis.

## 2. Materials and Methods

### 2.1. Samples Preparation

Sodium–vanadium oxide NaV_3_O_8_ was obtained by solid-state method. NH_4_VO_3_ (analytical grade) and Na_2_CO_3_ (reagent grade) were used as the starting components. The required amounts of the starting materials were weighed within ±0.0001 g (FX40-CJ analytical balance, Japan) and mixed together by grinding in a jasper mortar, then the mixture was heated in alundum crucible. The temperature for the final stage of sintering was chosen on the basis of DSC and TG data. Thermal analysis was performed using STA 449 F1 Jupiter instrument (NETZSCH, Selb, Germany) in alundum crucibles across a 35–580 °C temperature range at the heating rate of 10 °C/min. Measuring cell was blownthrow with air at arate of 20 mL/min. The data obtained were processed by means of NETZSCH Proteus software. At first, based on analysis of DSC and TG curves, we made a suggestion on the chemical reactions sequence at the starting mixture heating and temperature at which stabilization of the mass is observed was determined (539 °C). This was chosen as a minimal temperature of synthesis. Temperature increase increases rate of phase formation but on the other hand it can affect adversely on microstructure of material. For that reason we conducted anumber of isothermal heat treatments at different temperatures in 4–5 h with following X-ray analysis. Based on the results obtained, optimal temperature and time of sintering were chosen.

### 2.2. Samples Characterization

The phase composition of the samples was monitored by X-ray diffraction analysis (**XRD**) on a Rigaku DMAX-2200 diffractometer (Rigaku, Tokyo, Japan) in filtered Cu *Kα*-radiation generated at 40 kW, 30 mA (*λ* = 1.54178 Å) in the range of 2Ө = 15–70° stepwise with 0.3 s counting time and the step of 0.02°. Jade 6.5. Software was used to calculate unit cell parameters. The error of the cell parameters determining did not exceed 0.02%.

The Raman spectra were collected using U-1000 microscope-spectrometer (RENISHAW, Stonehouse, UK) (Ar^+^-radiation, *λ* = 514 nm) at 100–1200 cm^−1^.

The morphology of the synthesized material as well as the distribution and shape of its particles were studied using data of scanning electron microscopy (**SEM**) (MIRA 3 LMU TESCAN, Brno, Czech Republic). The particle-size distribution of the samples was studied using a particle-size analyzer Analysette 22 Nano Tec plus (Fritsch, Selb, Germany).

The ratio V^4+^/V^5+^ in NaV_3_O_8_ was determined by X-ray photoelectron spectroscopy (**XPS**). XPS spectra were collected using a Thermo Fisher Scientific X-ray photoelectron spectrometer (Thermo Scientific, Stonehouse, UK) with monochromatic Al-Kα radiation (hν = 1486 eV). The analyzed area diameter was 400 μm and the binding energy scale was corrected using C1s peak (284.8 eV).

### 2.3. Measurement of Conductivity

The conductivity of NaV_3_O_8_ was measured on sintered pellets 10 mm in diameter and 1–2 mm thick. The synthesized NaV_3_O_8_ powder with particles less than 50 μm in size was pressed in a stainless-steel die at 200–300 MPa and then sintered for 4–5 h at 400 °C in air. Ga-Ag paste applied on the opposite sides of the pellet was used as electrodes. The total conductivity of sample was determined using P-40X potentiostat-galvanostat (Elins, Chernogolovka, Russia) with AC measurements by means of impedance spectroscopy and with DC measurements by pulse potentiometry with time resolution of 2 μs followed by the extrapolation of polarization curves to the pulse time. In addition, the electronic component of conductivity was found using DC with blocking Au electrodes at 20–40 mV. The experiment was performed in the heating and cooling mode in several parallel measurements. The heating/cooling rate was 2 °C/min. The temperature experiment was determined by a platinum–platinum–rhodium thermocouple to an accuracy of ±0.5 °C. Isothermal extracts were made at each temperature. The results obtained at cooling/heating were in good agreement.

## 3. Results and Discussion

### 3.1. Synthesis of Sodium–Vanadium Oxide NaV_3_O_8_

**Figure 1:**
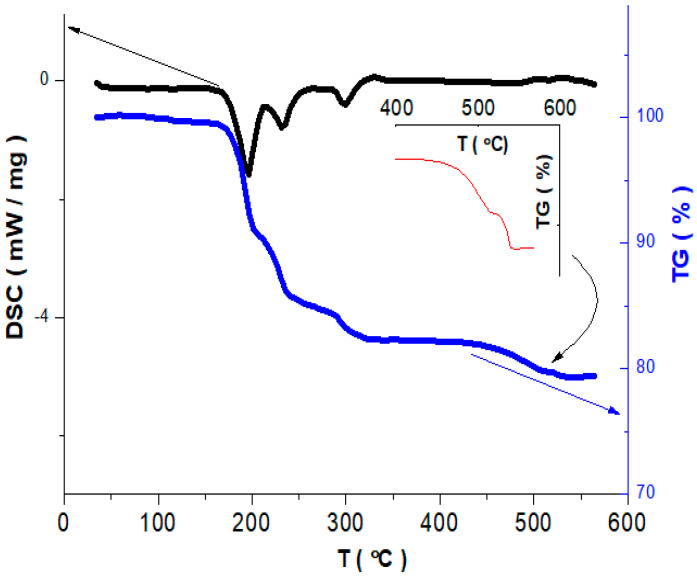
DSC and TG curves for the starting mixture of Na_2_CO_3_ and NH_4_VO_3_ (solid-state synthesis).

The conditions of synthesis were chosen based on the data of thermal analysis. Figure 1 contains DSC and TG curves for the starting mixture of NH_4_VO_3_ and Na_2_CO_3_. There are three peaks on the DSC curve in 200–300 °C temperature region. These peaks correspond to endothermic effects and a change of mass is observed on the TG curve at the corresponding temperatures. The first peak (~196 °C) is related to the start of thermal decomposition of NH_4_VO_3_. The change of mass that accompanies this process (9.54%) is in good agreement with the value (9.68%) calculated for the reaction describing the transformation of NH_4_VO_3_ into (NH_4_)_2_V_4_O_11_ according to (1).

4NH_4_VO_3_→(NH_4_)_2_V_4_O_11_ + 2NH_3_ + H_2_O (1)

The second peak corresponds to the further decomposition of (NH_4_)_2_V_4_O_11_ accompanied by the formation of ammonium polyvanadates, and the third one can be assigned to the decomposition of the latter with the formation of V_2_O_5_ [22]. Total mass loss at 350 °C determined from Figure 1 (17.5%) is close to the one calculated based on the total decomposition of NH_4_VO_3_ (19.3%). Above ~460 °C the mass of the reaction mixture again begins to decrease slowly, which is related to the release of CO_2_ after the interaction between Na_2_CO_3_ and V_2_O_5_, and at 539 °C the mass stabilizes (Figure 1, insert). The mass loss in the region of 460–540 °C was 5.85%, which is close to the one calculated according to the reaction (2), i.e., 6.77%.

Na_2_CO_3_ + 3V_2_O_5_→ 2NaV_3_O_8_ + CO_2_^↑^ (2)

In the case of the solid solution synthesis [21], the interaction of Na_2_CO_3_ and NH_4_VO_3_ took place primarily in the solution, and the thermogravimetric analysis of the reaction mixture obtained after the liquid was evaporated demonstrated that the mass stabilizes at ~350 °C, which allowed us to perform the synthesis at 380 °C. The temperature of solid-state synthesis should apparently lie between the temperature of mass stabilization (539 °C) and the temperature of incongruent melting of NaV_3_O_8_ (579 °C [23]). Therefore, the temperature of 565 °C was chosen for the synthesis of NaV_3_O_8_. The reaction mixture was cooled after 4–5 h and XRD analysis was carried out to monitor the phase composition during isothermal soaking. It was established that at 565 °C the interaction takes 10–12 h to complete. Based on these results, the temperature of 565 °C and the heat treatment time of 12 h were chosen as the optimum conditions for NaV_3_O_8_ synthesis.

**Figure 2:**
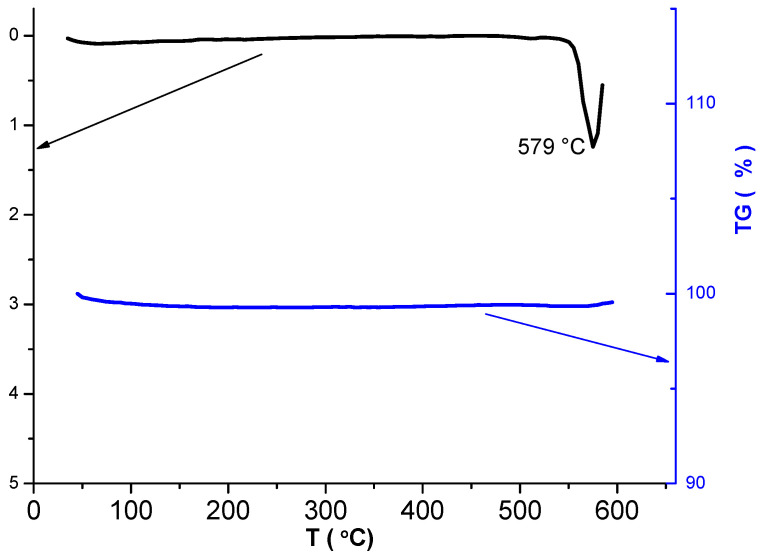
DSC and TG curves for NaV_3_O_8_.

The results of thermal analysis for NaV_3_O_8_ produced under such conditions are shown in Figure 2. One can see that the DSC curve contains one endothermic peak at 579 °C, which corresponds to the incongruent melting [23]. No thermal effects and mass changes have been recorded at lower temperatures. Thus, between the ambient temperature and the temperature of melting, the material obtained is thermally stable.

### 3.2. Phase Composition and Microstructure of NaV_3_O_8_

**Figure 3:**
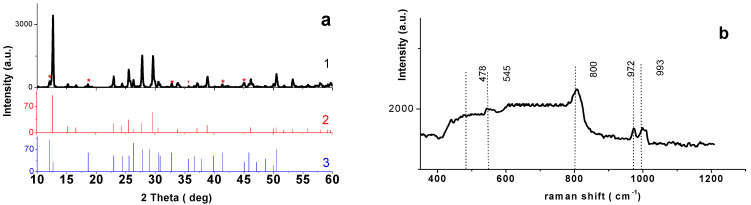
(**a**) XRD pattern of NaV_3_O_8_ (1) and line diagrams of powder pattern for NaV_3_O_8_ (PDF2, N° 28-1178) (2) and NaV_6_O_15_ (PDF2, N° 24-1155) (3); (**b**)Raman spectrum for NaV_3_O_8_.

The XRD powder pattern for the material obtained through solid-state synthesis is given in Figure 3a1. One can see that most reflections correspond to NaV_3_O_8_ phase, PDF2, 28-1171 (Figure 3a2), monoclinic structure, space group P21/m (11). Cell parameters a = 12.464(1) Å, b = 3.6098(5) Å, c = 7.3451(9) Å, α = γ = 90°, β = 107.36(6)° are in good agreement with the literature data [24]. In addition, the pattern contains some reflections of a lower intensity (<2%) indicated by an asterisk in Figure 3a1, which correspond to NaV_6_O_15_ PDF2, 24-1155 (Figure 3a3). The presence of this phase was previously reported in a number of other works discussing the synthesis of NaV_3_O_8_ [13,14]. NaV_6_O_15_ is also an electrochemically active component [25], but the authors [13,14] point out that the presence of small amounts of NaV_6_O_15_ alongside NaV_3_O_8_ has no effect on the electrochemical properties of the main phase.

The Raman spectrum for the synthesized sample (Figure 3b) is in good agreement with the literature data for NaV_3_O_8_. The characteristic bands observed at 993, 972, 795, 545, 478, 301, 227, 163, 134 cm^−1^ (marked by dotted lines in Figure 3b) practically coincide in their position and intensity with the ones given for NaV_3_O_8_ in [26]. According to the literature data, NaV_3_O_8_ and LiV_3_O_8_ are isostructural [23]. This lets us interpret that the bands in the Raman spectrum for the synthesized sample based on [26] and [27] were Raman spectra for LiV_3_O_8_ analyzed. As a result, one can conclude that the low-frequency bands (993 and 972 cm^−1^) correspond to the deformation vibrations, while the medium- and high-frequency bands indicate both the deformation vibrations of O-V-O, V-O-V groups, and the stretch vibrations of V-O bonds.

**Figure 4:**
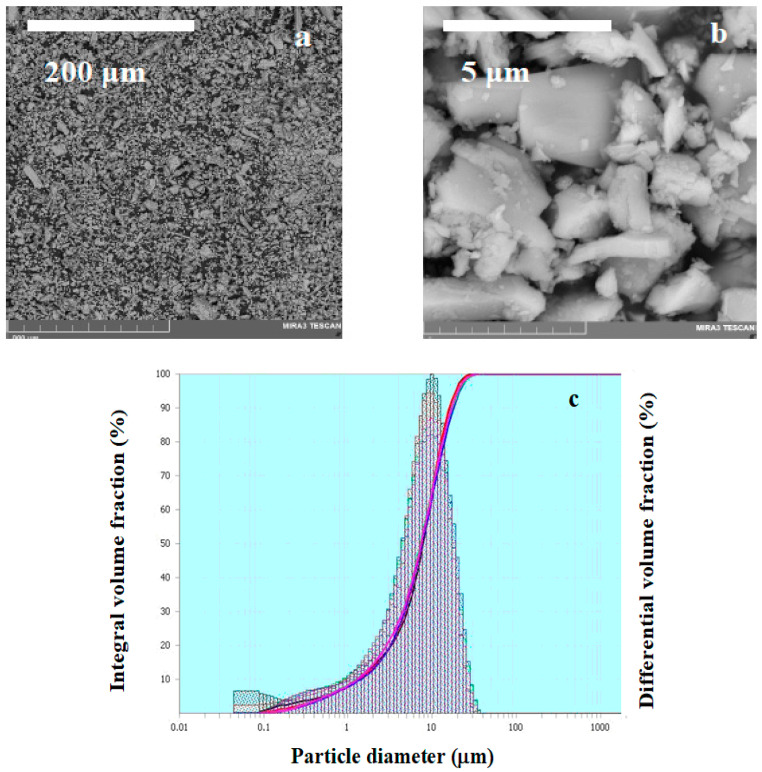
SEM image of NaV_3_O_8_ sample with different resolutions, (**a**) 500x and (**b**) 20.3kx; (**c**) particle size distribution in NaV_3_O_8_ samples obtained by solid state (**c**).

The microstructure of the sodium–vanadium oxide sample produced by solid-state synthesis is shown in Figure 4. The SEM image (Figure 4a,b) and particle size distribution in NaV_3_O_8_ samples (Figure 4c) shows that the grain size of the synthesized powder is 1–10 μm. The authors [16] synthesized NaV_3_O_8_ by sintering the mixture of Na_2_CO_3_ and V_2_O_5_ at 400, 500 and 600 °C for 12 h and report that synthesis at the lowest temperature (400 °C) yields the product with the smallest grain size (1–7 μm) characterized by fast intercalation/deintercalation of sodium cations. However, we obtained a powder with practically the same grain size at 565 °C (Figure 4). On the other hand, NaV_3_O_8_ produced via aqueous solution followed by heat treatment under conditions similar to the ones applied in [16] (380 °C, 12 h.) had grain size of about 100 nm [21]. Thus, prior interaction of Na_2_CO_3_ and NH_4_VO_3_ in an aqueous solution affects the grain size of NaV_3_O_8_ more than the final heat treatment temperature does.

### 3.3. Determining the Content of V^4+^ Ions in the Synthesized NaV_3_O_8_; Conductivity Measurement

**Figure 5:**
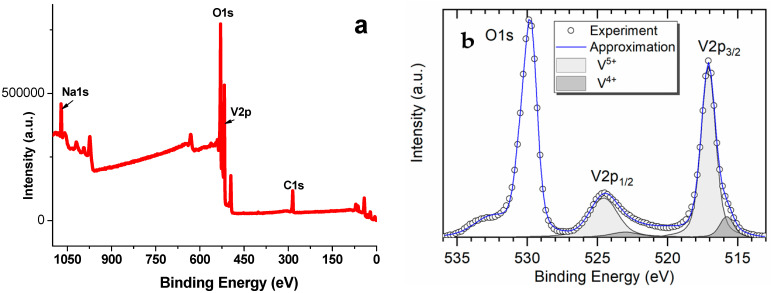
(**a**) Survey XPSspectrum forNaV_3_O_8_; (**b**) high-resolution XPS spectrum for V 2p inNaV_3_O_8_.

Successful application of a compound as active cathode material in a power source also depends on its electronic conductivity, which, in the case of oxide vanadium compounds, is determined by the ratio of V^4+^ and V^5+^ [28]. In view of this, investigating the influence of NaV_3_O_8_ synthesis technique on V^4+^/V^5+^ ratio is, no doubt, of some interest. The presence and amount of V^4+^ in the material synthesized was determined using XPS. The survey XPS spectrum for NaV_3_O_8_ (Figure 5a) was typical for this compound [29]. The spectrum contains clearly identifiable peaks of Na 1s at 1070 eV, C 1s at 285 eV, V 2p at 517 eV, O 1s at 530 eV (Figure 5a).

The high-resolution XPS spectrum for V 2p (Figure 5b) contains clearly identifiable peaks at 517.15 and524.48 eV, which can be ascribed to the spin-orbit splitting of V^5+^ 2p3/2 and V^5+^ 2p1/2, respectively [30]. Besides, there are peaks at 515.63 eV (V^4+^ 2p3/2) and 523.13 eV (V^4+^ 2p1/2) assigned to V^4+^. The content of V^4+^ and V^5+^, calculated from the areas of the peaks, was found to be 8% and 92%, respectively. Synthesis of NaV_3_O_8_ via aqueous solution yielded the values of 7% and 93% for V^4+^ and V^5+^, respectively [21]. A slightly higher content of V^4+^ in the case of solid-state synthesis may be related to partial reduction of V^5+^ to V^4+^ by the ammonia released during thermal decomposition of NH_4_VO_3_ [22].

**Figure 6:**
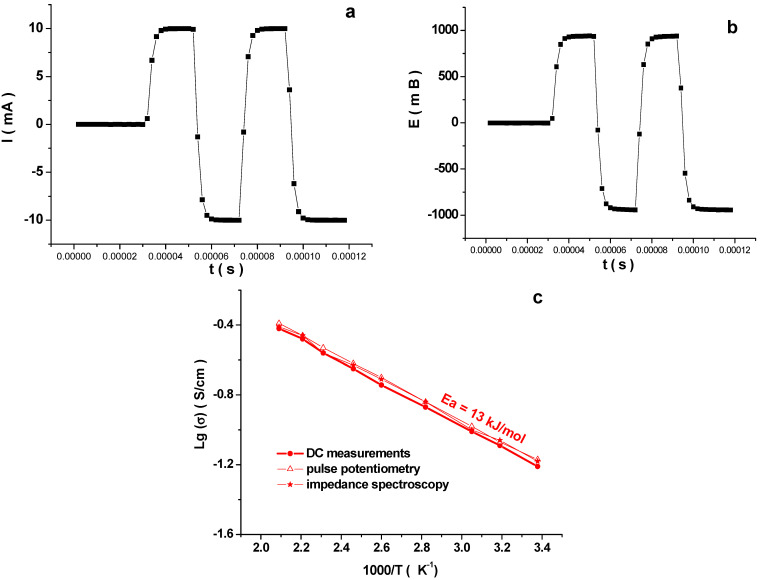
Results of measuring the resistance of the Ga-Ag│NaV_3_O_8_│ Ga-Ag cell by pulsepotentiometry. (**a**) Current pulse, (**b**) voltage response; t = 25 °C. (**c**) Temperature dependences of conductivity for NaV_3_O_8_ obtained by solid-state synthesis.

The electrical conductivity for NaV_3_O_8_ was determined using DC and AC conductivity measurements. The impedance spectroscopy measurements showed that the conductivity of the NaV_3_O_8_ samples obtained by solid state does not depend on the frequency. The total conductivity of polycrystalline NaV_3_O_8_ sample according to AC impedance spectroscopy measurements practically coincided with the value of conductivity yielded by DC measurements with blocking electrodes, which indicates that across the investigated temperature range, NaV_3_O_8_ is a chiefly electronic conductor. In addition, the electrochemical cell of the Ga-Ag│NaV_3_O_8_│Ga-Ag was studied by pulse potentiometry in order to determine the electrical resistance of the NaV_3_O_8_ sample. First, according to the voltage curves from the current, the maximum permissible load current for this experiment was determined, which was 10 mA. The measurement was performed in two pairs of pulses, +10 mA and −10 mA, with a reference value of 0 mA. The duration of each pulse was 20 μs. The resistance was calculated from the first points of each pulse. In Figure 6a,b is given experimental curves recorded at 25 °C. Pulse potentiometry measurements of conductivity resulted in the value similar to the one obtained by the two above-described methods. Thus, the results of the three measurement techniques practically coincide. Therefore, conductivity of NaV_3_O_8_ obeys the Arrhenius equation (Figure 6c) and the activation energy for the sample produced via solid-state synthesis is 13 kJ/mol.

The conductivity at room temperature was found to be 6.3 × 10^−2^ S × cm^−1^. This value is slightly higher than in the case of the sample obtained by aqueous solution technique (3.2 × 10^−2^ S × cm^−1^ [21]), which correlates with the higher content of V^4+^. Similar results were observed in the experiments with LiV_3_O_8_ [31]. The conductivity of the LiV_3_O_8_ sample synthesized via solid-state reaction was 7.9 × 10^−2^ S × cm^−1^ at 20 °C, the value for the sample produced via the method of aqueous solution was 2.5 × 10^−2^ S × cm^−1^ (V^4+^ content is 5% and 3%, respectively). Data on the physico-chemical properties of the sample NaV_3_O_8_ produced by solid-state synthesis obtained in this work and sample NaV_3_O_8_ produced by aqueous solution technique [21] comparedto similar sample LiV_3_O_8_ [31] are shown in Table 1. Generally, however, the conductivity values for LiV_3_O_8_ and NaV_3_O_8_ are quite close.

According to the literature data, LiV_3_O_8_ and NaV_3_O_8_ are isostructural and the only difference between them is the size of the interlayer spacing between vanadium–oxygen layers, where alkali cations are located [32,33]. The largeness of this space affects the mobility of alkali ions, while electronic conductivity is determined by vanadium–oxygen layers, whose structure is the same in both vanadates. Thus, it is reasonable to expect that if the ratios of V^4+^ and V^5+^ for LiV_3_O_8_ and NaV_3_O_8_ are close, the two compounds will have close values of electronic conductivity, which is the case.

## 4. Conclusions

Sodium–vanadium oxide NaV_3_O_8_, which is an attractive cathode material for SIBs, was synthesized via solid-state reaction between NH_4_VO_3_ and Na_2_CO_3_ at 565 °C. The product was characterized by XRD, thermal analysis and Raman spectroscopy. The morphology, shape and size of its particles and the ratio of V^4+^ and V^5+^ were studied, and AC and DC conductivity measurements were performed. The results were compared with the characteristics of the material having the same composition but produced by the reaction of NH_4_VO_3_ and Na_2_CO_3_ in a water solution followed by evaporation and heat treatment at 380 °C (aqueous solution method).

The material obtained through solid-state reaction contains a small amount of NaV_6_O_15_ alongside the monoclinic phase of NaV_3_O_8_. The vanadate is thermally stable between the ambient temperature and the temperature of melting; consequently, it can be used in appliances operating at elevated temperatures. It also has a high electronic conductivity (6.3 × 10^−2^ S × cm^−1^ at room temperature). The high electronic conductivity is an advantage if the vanadate under discussion is proposed as a cathode material for sodium power sources. The conductivity of NaV_3_O_8_ synthesized by the method of aqueous solution is twice smaller, thus, at first sight, the vanadate produced in this way is an inferior material compared to its counterpart produced by solid-state reaction. However, the conductivity of NaV_3_O_8_ altogether is not very high, and in cathodes of actual power sources it should apparently be combined with another species, characterized by a higher conductivity, e.g., carbon (acetylene black), which is usually used for this purpose. In this case, the difference in the conductivity of the materials obtained via solid-state synthesis and the method of aqueous solution is not important, while the particle size becomes essential. The solid-state technique yields the product with the grain size of 1–3 μm, while using the method of aqueous solution one can obtain a homogeneous nanostructured material with the average grain size of nearly 100 nm. According to the literature data, nanostructured cathode materials considerably improve the kinetics of alkali cations intercalation/deintercalation, and increase the capacity of power sources and their cyclability and coulombic efficiency. Therefore, the method of aqueous solution is a better alternative for the production of NaV_3_O_8_ compared to the solid-state technique.

## Figures and Tables

**Table 1:** Data on the physico-chemical properties of the sample NaV_3_O_8_ produced by solid-state synthesis obtained in this work and sample NaV_3_O_8_ produced by aqueous solution technique [21] compared to similar sample LiV_3_O_8_ [31].

Physico-Chemical Properties	Composition			
	NaV_3_O_8_ Solid-State Synthesis	NaV_3_O_8_ Aqueous Solution Technique [21]	LiV_3_O_8_ Solid-State Synthesis [31]	LiV_3_O_8_ Aqueous Solution Technique [31]
particle size	1–10 μm	100 nm	1–10 μm	200 nm
conductivity, S × cm^−1^	6.3 × 10^−2^	3.2 × 10^−2^	7.9 × 10^−2^	2.5 × 10^−2^
V^4+^ content, %	8	7	5	3

## Data Availability

The data presented in this study are available on request from the corresponding author.
